# Mobilization of Copper ions by Flavonoids in Human Peripheral Lymphocytes Leads to Oxidative DNA Breakage: A Structure Activity Study

**DOI:** 10.3390/ijms161125992

**Published:** 2015-11-09

**Authors:** Hussain Arif, Nida Rehmani, Mohd Farhan, Aamir Ahmad, Sheikh Mumtaz Hadi

**Affiliations:** 1 Department of Biochemistry, Faculty of Life Sciences, Aligarh Muslim University, Aligarh 202002, UP, India; arifkap@gmail.com (H.A.); nida.rehmani4@gmail.com (N.R.); farhan@mohdfarhan.com (M.F.); 2 Karmanos Cancer Institute, Wayne State University School of Medicine, Detroit, MI 48201, USA

**Keywords:** flavonoids, comet assay, structure activity relationship, molecular docking, Isothermal Titration Calorimetric Measurements, hydroxyl group positions

## Abstract

Epidemiological studies have linked dietary consumption of plant polyphenols with lower incidence of various cancers. In particular, flavonoids (present in onion, tomato and other plant sources) induce apoptosis and cytotoxicity in cancer cells. These can therefore be used as lead compounds for the synthesis of novel anticancer drugs with greater bioavailability. In the present study, we examined the chemical basis of cytotoxicity of flavonoids by studying the structure–activity relationship of myricetin (MN), fisetin (FN), quercetin (QN), kaempferol (KL) and galangin (GN). Using single cell alkaline gel electrophoresis (comet assay), we established the relative efficiency of cellular DNA breakage as MN > FN > QN > KL > GN. Also, we determined that the cellular DNA breakage was the result of mobilization of chromatin-bound copper ions and the generation of reactive oxygen species. The relative DNA binding affinity order was further confirmed using molecular docking and thermodynamic studies through the interaction of flavonoids with calf thymus DNA. Our results suggest that novel anti-cancer molecules should have ortho-dihydroxy groups in B-ring and hydroxyl groups at positions 3 and 5 in the A-ring system. Additional hydroxyl groups at other positions further enhance the cellular cytotoxicity of the flavonoids.

## 1. Introduction

Development of cancer is a complex process that involves factors that are important in the significant steps of initiation, promotion and progression, resulting in the uncontrolled proliferation and growth of cancer cells. It is now believed that dietary factors from various plants can modulate the phenomenon of carcinogenesis, thus relating the food stuffs to benefits beyond the basic nutritional requirements [1,2,3]. Among such dietary constituents, plant polyphenols are considered to be the most effective in cancer chemoprevention in humans [4,5]. Flavonoids can be classified under 14 different categories such as flavonols, flavones, flavanones, and others [6,7]. Structurally, flavonoids are C6–C3–C6 compounds consisting of two benzene rings, joined by the C3 aliphatic chain with a heterocyclic pyran ring [8]. Flavonoids are a subclass of plant polyphenols that include flavonols like myricetin, fisetin, quercetin, kaempferol and galangin. The mechanism by which these anticancer compounds are able to inhibit proliferation and induce apoptosis in cancer cells is not very clearly understood and has been investigated with profound interest. In recent years, a number of reports have suggested that a number of these flavonoids including myricetin, fisetin, apigenin quercetin and kaempferol induce apoptotic cell death in cancer cell lines [9,10,11,12]. One particularly interesting observation is that a number of these flavonoids induce apoptosis and affect tumorigenesis in cancer cells but not in normal cells [13,14].

Previous reports from our laboratory have established that many classes of plant-derived polyphenolic compounds, including flavonoids, can bring about oxidative strand breakage in isolated DNA or cellular DNA either alone or in the presence of transition metal ions such as copper [15,16]. It is well established that copper is an important and the most redox active metal ion when compared to the other metal ions that are present in biological systems [17]. Copper is found in chromatin, closely associated with DNA bases, particularly the guanine base [18]. Most flavonoids possess both prooxidant as well as antioxidant properties [19,20,21]. Earlier, we proposed that the prooxidant cytotoxic action of flavonoids might be a very important mechanism, leading to the anticancer and apoptosis-inducing ability [5,22]. We proposed that such prooxidant mechanism against cancer cells involves mobilization of endogenous copper ions [5]. Among the metal ions, the concentration of iron is the highest in cells, whereas copper and zinc are the major metals present in the nucleus [18,23]. Copper is known to possess highest redox activity which facilitates its rapid recycling, in the presence of compounds such as plant polyphenols and molecular oxygen, leading to generation of reactive oxygen species (ROS) such as the hydroxyl radical. It may be mentioned that chromatin-bound copper can be mobilized by copper chelators such as 1,10-phelanthroline to cause internucleosomal DNA fragmentation [24]. A number of studies suggest that serum [25,26], tissue [27] and cellular copper levels are particularly high in various cancers [28]. Such a mechanism is not dependent on receptor or mitochondria mediated programmed cell death [29]. Over the years, we have validated our hypothesis with considerable success [3,5,17,22,30]. Specifically, we have shown that oxidative cellular DNA breakage by polyphenols involves mobilization of nuclear copper [30]. Moreover, overload of copper in lymphocytes results in increased polyphenol-mediated cellular DNA degradation [31]. Finally, we have also successfully demonstrated that polyphenols can inhibit growth of multiple cancer types in culture; this effect is reduced significantly in the presence of copper specific chelators, with chelators of iron and zinc relatively ineffective [32,33,34].

**Figure 1:**
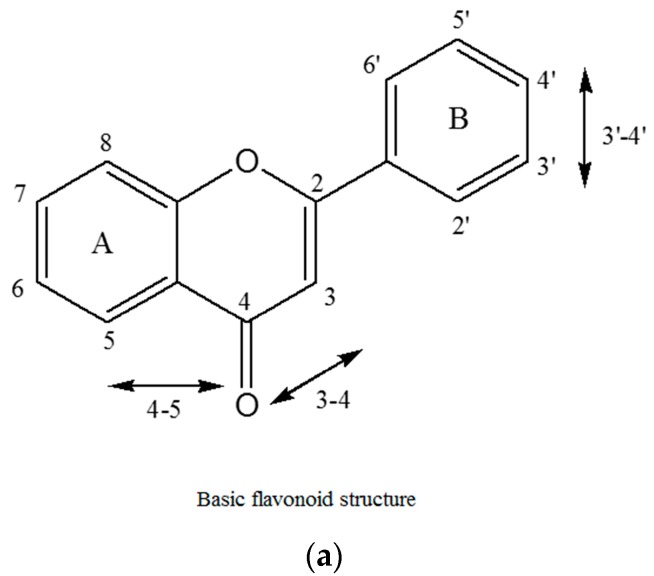

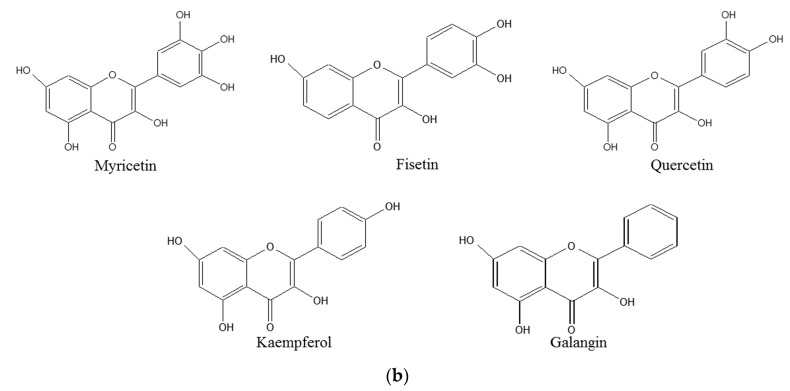
(**a**) Basic flavonoid structure showing the A and B rings and the conventional numbering of carbon atoms and (**b**) structure of various flavonoids used in this study.

Plant polyphenols including flavonoids are rapidly metabolized in animal cells and thus have poor bioavailability [35]. However, as there is considerable literature on the anticancer properties of flavonoids, it is reasonable to assume that flavonoids can be exploited as lead compounds for the synthesis of novel compounds with enhanced bioavailability. Taking this into consideration, we assessed the chemical basis of the cytotoxic activity of different flavonoids (myricetin, fisetin, quercetin, kaempferol, galangin) and evaluated the structure-activity relationship (SAR) of these compounds. For example, we show that the number and position of hydroxyl groups in the B-ring of flavonoid skeleton (Figure 1a) is important in determining the cellular DNA degrading capacity of these compounds. The structures of various flavonoids used in this study are shown in Figure 1b.

## 2. Results and Discussion

### 2.1. Cellular DNA Breakage by Flavonoids in Intact and Permeabilized Lymphocytes as Measured by Comet Assay

Comet assay (single-cell gel electrophoresis) is well known tool for the quantification of DNA damage in cellular systems. [36]. A modified comet assay, using alkaline conditions, made it possible to detect the single-strand breaks as well as the alkaline-labile sites in DNA [37]. In this assay, the two single-strand breaks that are closely opposed, show up as a double strand break. In our previous work, we were able to demonstrate that the comet assay can be used to measure the cellular DNA degradation induced by polyphenols in human peripheral lymphocytes [38]. This has been confirmed by reports from other laboratories as well [39] where comet assay was used to evaluate reactive oxygen species (ROS)-mediated DNA breakage induced by estrogen-like compounds, diethylstilbestrol, diadzein and genistein. Here, we used the comet assay to test DNA breakage by 100 µM concentrations of MN, FN, QN, KL and GN in isolated lymphocytes. As clear from Table 1, we observed a prominent increase in the GN–, KL–, QN–, FN– and MN–mediated DNA breakage, as shown by increased comet tails. It is seen that the highest level of DNA degradation is caused by myricetin followed by fisetin, quercetin, kaempferol and galangin. According to our hypothesis, polyphenols mobilize chromatin bound copper directly, resulting in cellular DNA breakage. We, therefore, tested the effect of same concentrations of flavonoids on permeabilized lymphocytes (Table 1). We used permeabilized lymphocytes because permeabilization allows for a direct entry and interaction of flavonoids with the cell nuclei, resulting in significantly increased DNA breakage. As seen in the table this indeed is found to be the case. Table 1 clearly suggests that the length of comet tail was greater in permeabilized lymphocytes, as compared to intact cells, thus further supporting the notion that test compounds interact better with nuclei in a permeabilized system. We also observed that in both intact and permeabilized cells the rate of DNA breakage was greatest for myricetin, followed by fisetin, quercetin, kaempferol and galangin respectively.

**Table 1:** A comparison of DNA breakage induced by various flavonoids in intact lymphocytes and permeabilized lymphocytes as a function of comet tail lengths. Values reported are ±SEM of three independent experiments. *p* < 0.01 by comparison with control (untreated cells) for each compound.

Flavonoids (100 µM)	Comet Tail Length (µM)	
	Intact Cells	Permeabilized Cells
Control (No Flavonoid)	3.95 ± 0.19	4.06 ± 0.20
Myricetin	25.06 ± 1.25	28.90 ± 1.44
Fisetin	20.45 ± 1.02	25.03 ± 1.25
Quercetin	17.74 ± 0.88	23.50 ± 1.17
Kaempferol	16.03 ± 0.80	20.66 ± 1.03
Galangin	14.85 ± 0.74	19.44 ± 0.97

### 2.2. Formation of Complexes of Flavonoids

**Figure 2:**
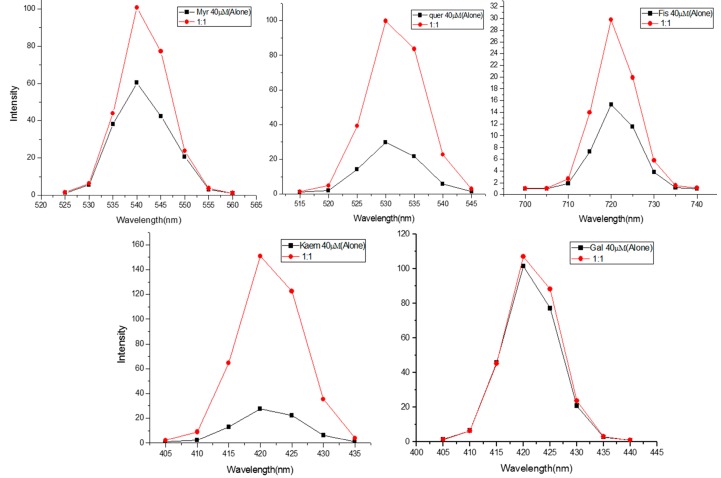
Effect of increasing native DNA base pair molar ratio on the fluorescence emission spectra of Flavonoids. Flavonoids were excited at different wavelengths (MN = 380 nm, FN = 365 nm, QN = 370 nm, KL = 365 nm, GN = 365 nm) in the presence of increasing native DNA base pair molar (1:1) and the emission spectra were recorded.

Figure 2 shows the effect of addition of calf thymus DNA to MN, FN, QN, KL and GN at an equimolar base pair ratio of DNA *vs.* flavonoids (1:1). The mixture was excited at specific wavelengths provided in legends. It is seen that there is an enhancement of the emission spectra of all the five flavonoids in the presence of DNA. However, we did not notice a shift in λ_max_ of emission. This suggests a simple mode of binding of flavonoids to DNA.

### 2.3. Effect of Reactive Oxygen Scavengers on the Flavonoids-Induced DNA Breakage in Permeabilized Lymphocytes

In order to confirm that the flavonoid-induced lymphocyte DNA breakage results from ROS generation, we have studied the effect of some scavengers of ROS on comet tail length formation by plant polyphenols [5]. Table 2 summarizes the observations from an experiment where we tested the effects of catalase, SOD and thiourea on flavonoid-induced DNA degradation in permeabilized lymphocytes. As expected, all the flavonoids induced significant DNA damage, and resulted in increased tail lengths of comets, compared to control cells (*p* < 0.01). The three scavengers tested (catalase, SOD and thiourea) were found to particularly inhibit flavonoid-induced DNA breakage very effectively, as suggested by reduced comet tail length. Catalase and SOD are scavengers of H_2_O_2_ and superoxide, respectively, while thiourea scavenges hydroxyl radicals. From the experiment presented here, we can safely infer that ROS is primarily involved in the cellular damage caused by flavonoids and that the main ROS involved in the process are the superoxide anions and the hydroxyl radicals. It is worth mentioning that superoxide anion spontaneously leads to the formation of H_2_O_2_. H_2_O_2_, in turn, leads to the generation of hydroxyl radicals through a process that is dependent on the oxidation of reduced transition metals [40]. It is also well recognized that the hydroxyl radicals interact with DNA in such proximity that a complete scavenging of hydroxyl radicals is not achievable [41].

**Table 2:** Effect of scavengers of reactive oxygen species on flavonoid-induced cellular DNA breakage in permeabilized cells. * *p* < 0.05, compared with control (flavonoid only) cells. Data represent ±SEM of three independent experiments.

Treatment	Intact Cells		Permeabilized Cells	
	Comet Tail Length %	Inhibition (µM)	Comet Tail Length %	Inhibition (µM)
Control	3.156 ± 0.17	-	3.25 ± 0.16	-
Myricetin (100 µM)	25.03 ± 1.25	-	31.56 ± 1.57	-
+SOD (100 µg/mL)	11.26 ± 0.56 *	55.0	13.28 ± 0.76 *	57.9
+Catalase (100 µg/mL)	12.46 ± 0.62 *	50.2	15.05 ± 0.80 *	52.3
+Thiourea (1 mM)	14.08 ± 0.70 *	43.7	16.86 ± 0.84 *	46.5
Fisetin (100 µM)	22.96 ± 1.14	-	27.04 ± 1.35	-
+SOD (100 µg/mL)	12.20 ± 0.61 *	46.8	14.24 ± 0.71 *	47.3
+Catalase (100 µg/mL)	12.76 ± 0.63 *	44.4	14.64 ± 0.73 *	45.8
+Thiourea (1 mM)	13.04 ± 0.65 *	43.2	15.05 ± 0.75 *	44.3
Quercetin (100 µM)	20.32 ± 1.01	-	24.54 ± 1.22	-
+SOD (100 µg/mL)	10.46 ± 0.52 *	48.5	11.23 ± 0.56 *	54.2
+Catalase (100 µg/mL)	11.06 ± 0.55 *	45.5	13.42 ± 0.67 *	45.3
+Thiourea (1 mM)	14.80 ± 0.74 *	27.1	14.95 ± 0.74 *	39.07
Kaempferol (100 µM)	17.63 ± 0.88	-	21.54 ± 1.07	-
+SOD (100 µg/mL)	9.50 ± 0.47 *	46.1	10.36 ± 0.51 *	51.9
+Catalase (100 µg/mL)	10.44 ± 0.52*	40.7	10.45 ± 0.52 *	51.4
+Thiourea (1 mM)	11.05 ± 0.55 *	37.3	12.45 ± 0.62 *	42.2
Galangin (100 µM)	14.40 ± 0.72	-	18.44 ± 0.92	-
+SOD (100 µg/mL)	8.55 ± 0.32 *	40.6	9.65 ± 0.63 *	47.6
+Catalase (100 µg/mL)	9.40 ± 0.37 *	34.7	11.32 ± 0.59 *	38.6
+Thiourea (1 mM)	10.15 ± 0.50*	29.5	10.55 ± 0.53 *	42.7

### 2.4. Effect of Specific Chelators of Metals on Flavonoid-Induced DNA Breakage in Intact and Permeabilized Lymphocytes

Next we used the specific chelators of several metals (such as the chelators that selectively bind to copper, iron and zinc) and evaluated their effect, if any, on flavonoid-induced DNA degradation in whole as well as permeabilized cells. We observed a clear inhibition of DNA degradation in whole cells when we used neocuproine. Neocuproine is a Cu(I) chelator that is cell membrane permeable (Table 3). However, we did not see any inhibition when we used bathocuproine. Bathocuproine is a water soluble analog of neocuproine, but is membrane-impermeable. Similarly, no inhibition was observed when we used desferrioxaminemesylate (Fe(II)-specific chelator) and histidine (a zinc-specific chelator). In permeabilized lymphocytes, it was observed that both the Cu(I) chelators, neocuproine and bathocuproine, could inhibit DNA breakage because membrane permeability was no longer a determining factor. Iron and zinc chelators were still found to be ineffective in affording protection.

**Table 3:** Effect of chelators of metals on flavonoids-induced cellular DNA degradation in lymphocytes. Values shown in this table represent flavonoid-induced cellular DNA breakage in whole and permeabilized lymphocytes measured as a percentage of the control (DNA breakage in the absence of chelators). Data represent ±SEM of three independent experiments. * *p* < 0.05, compared to untreated whole lymphocytes (comet tail length = 3.43 ± 0.13). ** *p* < 0.05, compared to untreated permeabilized lymphocytes (comet tail length = 3.85 ± 0.17).

Dose	Whole Lymphocytes		Permeabilized Lymphocytes	
	Comet Tail Length %	of Control (µM)	Comet Tail Length %	of Control (µM)
**Myricetin** (200 µM)	31.24 ± 1.56 *	-	37.90 ± 1.89 **	-
+Neocuproine (200 µM)	21.06 ± 1.12	32.5	23.04 ± 1.15	39.2
+Bathocuproine (200 µM)	29.86 ± 1.50	4.41	22.50 ± 1.12	40.6
+Histidine (200 µM)	30.63 ± 1.55	1.95	37.73 ± 1.88	0.44
+Desferioxaminemesylate (200 µM)	30.04 ± 1.53	3.84	37.15 ± 1.85	1.97
**Fisetin** (200 µM)	29.80 ± 1.49 *	-	36.04 ± 1.80 **	-
+Neocuproine (200 µM)	20.37 ± 0.91	31.6	23.54 ± 1.17	34.68
+Bathocuproine (200 µM)	28.84 ± 1.39	3.22	22.33 ± 1.11	38.04
+Histidine (200 µM)	29.40 ± 1.47	1.34	36.00 ± 1.80	0.11
+Desferioxaminemesylate (200 µM)	29.11 ± 1.45	2.31	35.38 ± 1.76	1.83
**Quercetin** (200 µM)	26.33 ± 1.40 *	-	33.34 ± 1.66 **	-
+Neocuproine (200 µM)	18.54 ± 0.86	29.5	21.96 ± 1.09	34.13
+Bathocuproine (200 µM)	25.69 ± 1.31	2.43	21.43 ± 1.07	35.72
+Histidine (200 µM)	26.04 ± 1.35	1.10	33.30 ± 1.66	0.11
+Desferioxaminemesylate (200 µM)	26.00 ± 1.34	1.25	32.89 ± 1.64	1.34
**Kaempferol** (200 µM)	21.76 ± 1.18 *	-	30.23 ± 1.51 **	-
+Neocuproine (200 µM)	16.53 ± 0.72	24.03	20.44 ± 1.02	32.38
+Bathocuproine (200 µM)	21.28 ± 1.15	2.20	19.70 ± 0.98	34.83
+Histidine (200 µM)	21.68 ± 1.17	0.36	30.19 ± 1.50	0.13
+Desferioxaminemesylate (200 µM)	21.52 ± 1.17	1.10	30.07 ± 1.50	0.52

### 2.5. Stoichiometry of Cu(II) Reduction by Flavonoids

**Figure 3:**
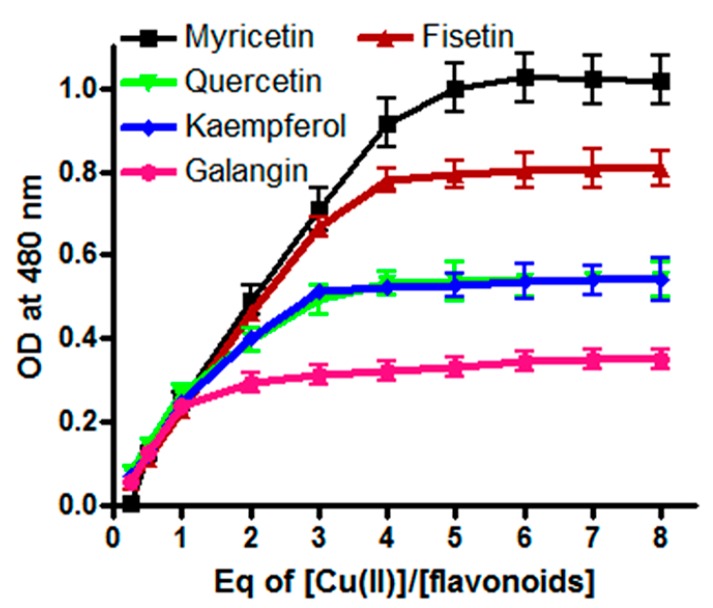
Stoichiometry of Flavonoid–Cu(II) interactions. The concentration of myricetin, fisetin, quercetin, kaempferol and galangin was 10 µM in the presence of 0.3 mM bathocuproine (10 mM Tris–HCl). All the points are representative of triplicate samples and mean values are plotted.

Redox recycling of Cu(II)/Cu(I) is known to be an important factor in the induction of copper-mediated DNA breakage [22,30]. Therefore, we next assessed the relative efficiency of reduction of Cu(II) by MN, FN, QN, KL and GN through stoichiometric studies. Jobplots of absorbance *versus* (Cu(II))/(flavonoids) (Figure 3) indicate that all flavonoids were able to reduce Cu(II) to Cu(I). However, it is also evident that myricetin was the most efficient reducer of Cu(II) among all the tested flavonoids whereas galangin was the least efficient.

### 2.6. Intracellular Generation of H_2_O_2_/ROS by Flavonoids

**Figure 4:**
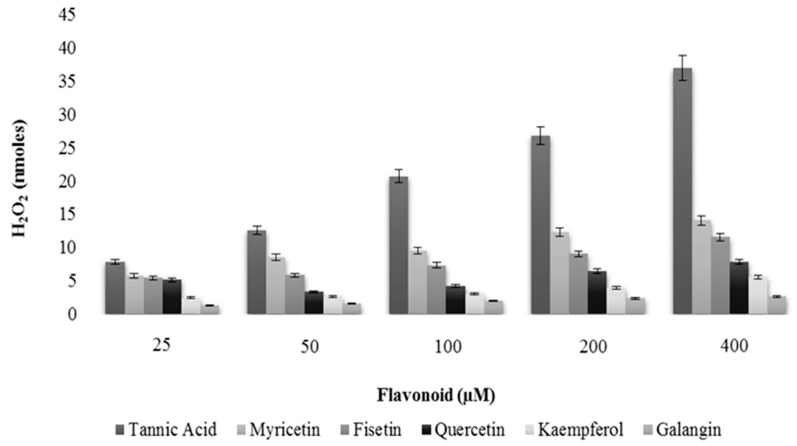
A comparison of the rate of H_2_O_2_ formation by tannic acid, myricetin, fisetin, quercetin, kaempferol and galangin in the incubation medium as determined by FOX assay.

It has been reported that polyphenols can auto-oxidize under *in vitro* conditions resulting in generation of H_2_O_2_ radicals and quinone, which can potentially enter cellular nuclei, thus causing damage to many molecules [42]. This interaction can further generate extraneous ROS accounting for even further damage to the integrity of DNA. In an attempt to exclude this possibility, we carried out our experiment for the generation of H_2_O_2_ by flavonoids in the incubation medium. We also used tannic acid in our experimental set-up, a positive control for H_2_O_2_ production [43]. As seen in Figure 4, tannic acid effectively generates H_2_O_2_. Our test compounds were clearly not as effective producers of H_2_O_2_ as tannic acid but we could still observe H_2_O_2_ production by flavonoids myricetin, fisetin and quercetin. H_2_O_2_ production by kaempferol and galangin was measured to be almost negligible, when compared to the other tested flavonoids and the tannic acid. Thus, these results support results described in Table 1 where the order of cellular DNA breakage by flavonoids was MN > FN > QN > KL > GN.

**Figure 5:**
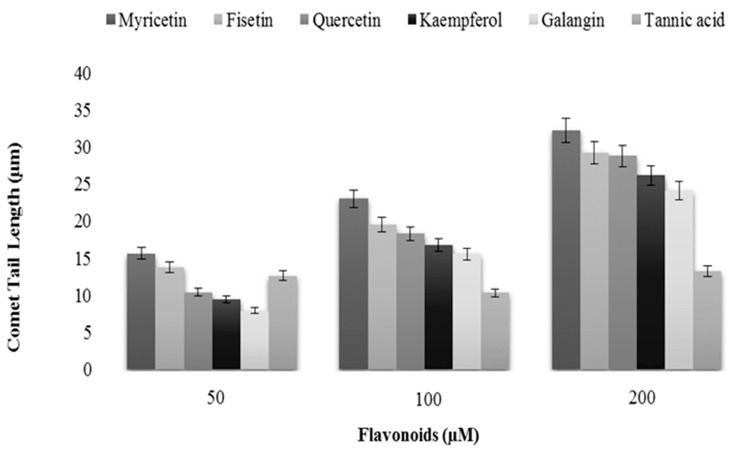
A comparison of DNA breakage induced by tannic acid, myricetin, fisetin, quercetin, kaempferol and galangin in human peripheral lymphocytes as a function of comet tail lengths. Values reported are ±SEM of three independent experiments.

We also evaluated the length of comet tail as a function of increasing flavonoids concentrations. Again tannic acid was used as internal control. The results shown in Figure 5 clearly show that exposure to flavonoids resulted in the formation of comet tails, which were found to positively correlate with the increasing concentration of the flavonoids. Tannic acid, however, was not very effective in causing DNA damage, as visualized by increasing comet tail length, particularly at the highest dose tested. This shows that there is no clear correlation between H_2_O_2_ production and cellular DNA damage because the most effective generator of H_2_O_2_ did not cause the most DNA damage.

### 2.7. Thermodynamics of Flavonoid–ctDNA Binding-Iso Thermal Calorimetric Studies (ITC)

Thermodynamic parameters of flavonoid complexes with ctDNA were assessed by fitting the integrated heats as per the one binding site model shown in Table 4. Enthalpy changes for binding of five flavonoids to ctDNA were negative, indicating an exothermic binding process with electrostatic interactions. Further, the negative entropy terms, *T*∆*S*° = −2.08 kJ·mol^−1^, −8.34 kJ·mol^−1^, −14.6·kJ·mol^−1^, −17.28 kJ·mol^−1^ and −19.07 kJ·mol^−1^ for GN, KL, QN, FN and MN, respectively, indicate the binding of these flavonoids is predominately enthalpy driven. It can also be seen that for all flavonoids tested, both enthalpy as well as entropy have negative values, which suggests an essential role of hydrogen bonding and van der Waals force in the binding of flavonoids to ctDNA. During the interaction process, the higher negative values of ∆*G*° suggests that the formation of flavonoid–ctDNA complexes will be much more spontaneous in nature as compared to the complexes which exhibit lower negative values of ∆*G*°. Considering the ∆*G*° values for different flavonoids, it can be concluded that myricetin has the highest negative ∆*G*° value (−30.01 kJ·mol^−1^) and therefore will form stronger interactions with ctDNA as compared to the rest of the tested flavonoids.

**Table 4:** The binding constant/stoichiometry and thermodynamic parameters for the binding of myricetin, fisetin, quercetin, kaempferol and galangin to ctDNA, as determined with ITC at 25 °C. “*n*”, possible number of binding sites; *K*_a_, binding constant; ∆*H*, ∆*S* and ∆*G*, standard changes of enthalpy, entropy and Gibb’s free energy for per mole of respective flavonoid bound to ctDNA.

Flavonoid	*K*_a_( M^−1^)	*n*	∆*H* (kJ/mol)	∆*S* (J/mol/k)	∆*G* (kJ/mol)
Myricetin	1.9 × 10^2^	74.62	−49.21	−64.43	−30.01
Fisetin	0.9 × 10^2^	66.50	−43.01	−58.18	−25.67
Quercetin	0.8 × 10^2^	63.32	−39.81	−49.76	−24.98
Kaempferol	0.6 × 10^2^	51.43	−31.76	−28.89	−23.15
Galangin	0.3 × 10^2^	44.67	−22.25	−7.11	−20.13

**Figure 6:**
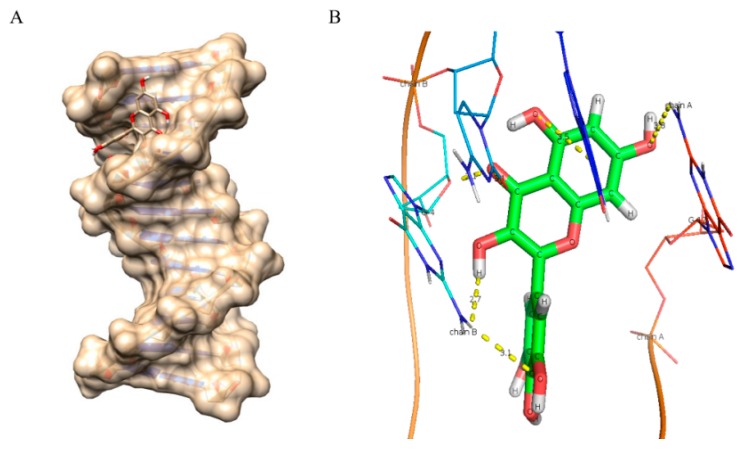
Molecular docked structure of myricetin with B-DNA. (**A**) Surface view interaction of myricetin with B-DNA; (**B**) Hydrogen bonding interactions (6) and distance in Å of myricetin with dodecamer duplex of sequence (CGCGAATTCGCG)_2_ (PDB ID: 1BNA).

### 2.8. Molecular Docking Studies

**Figure 7:**
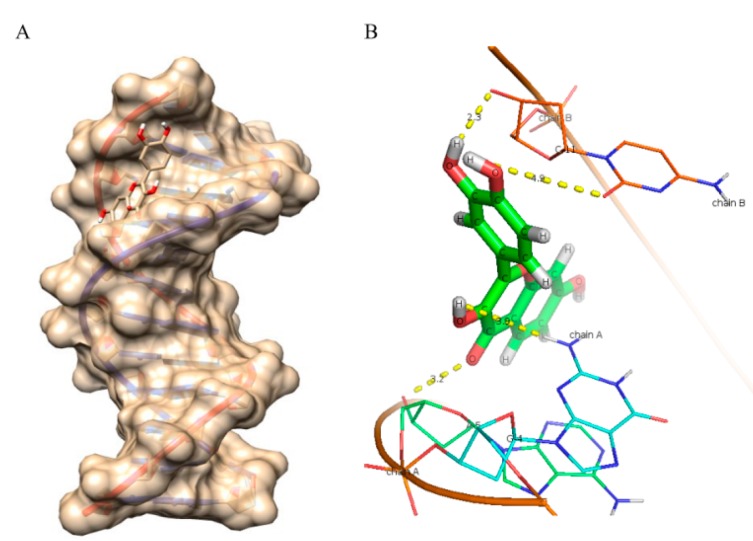
Molecular docked structure of fisetin with B-DNA. (**A**) Surface view interaction of fisetin with B-DNA; (**B**) Hydrogen bonding interactions (4) and distance in Å of fisetin with dodecamer duplex of sequence (CGCGAATTCGCG)_2_ (PDB ID: 1BNA).

**Figure 8:**
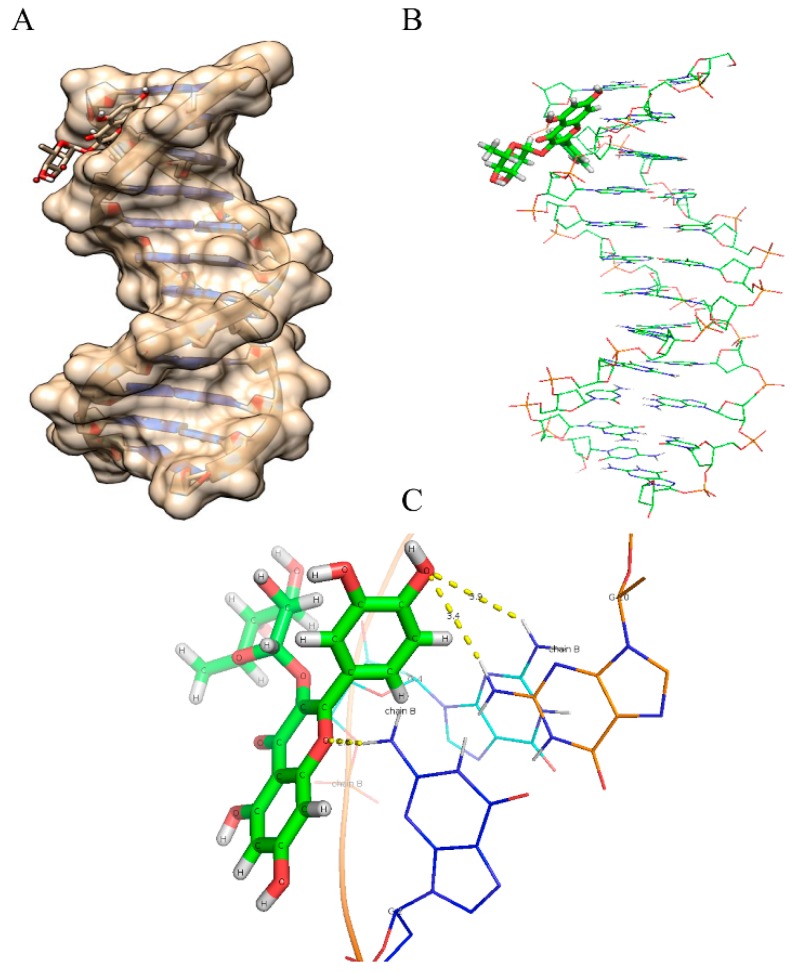
Molecularly docked structure of quercetin with B-DNA. (**A**) Surface view interaction of quercetin with B-DNA; (**B**) Line pose of quercetin showing the majority of its structure away from the minor groove nucleotides; (**C**) Hydrogen bonding interactions (3) and distance in Å of quercetin with dodecamer duplex of sequence (CGCGAATTCGCG)_2_ (PDB ID: 1BNA).

**Figure 9:**
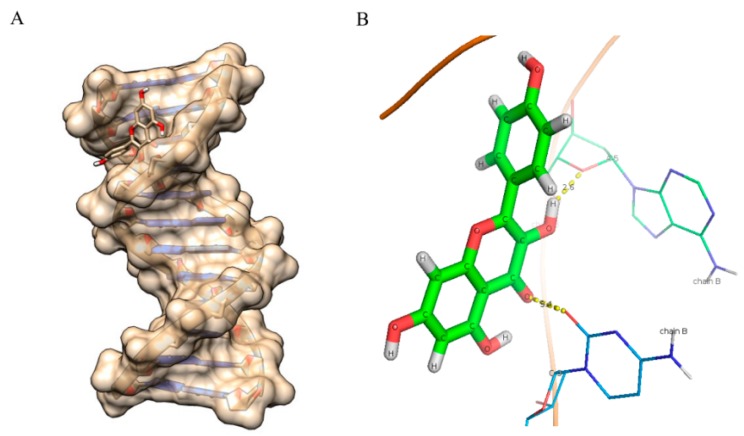
Molecularly docked structure of kaempferol with B-DNA. (**A**) Surface view interaction of kaempferol with B-DNA; (**B**) Hydrogen bonding interactions (2) and distance in Å of kaempferol with dodecamer duplex of sequence (CGCGAATTCGCG)_2_ (PDB ID: 1BNA).

**Figure 10:**
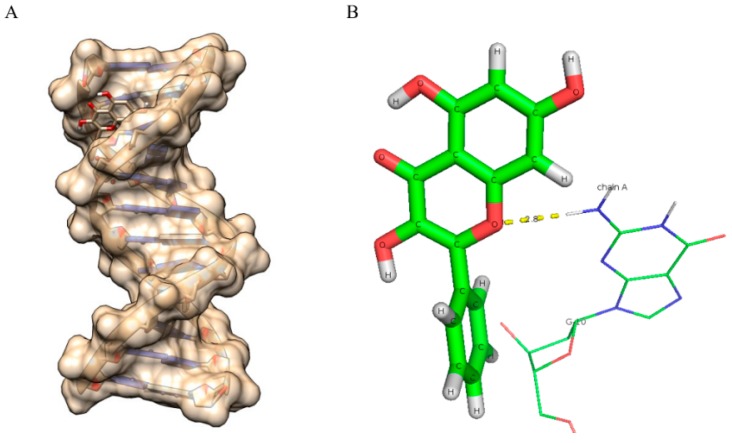
Molecularly docked structure of galangin with B-DNA. (**A**) Surface view interaction of galangin with B-DNA; (**B**) Hydrogen bonding interactions (1) and distance in Å of galangin with dodecamer duplex of sequence (CGCGAATTCGCG)_2_ (PDB ID: 1BNA).

As evident from the results (Figure 6, Figure 7, Figure 8, Figure 9 and Figure 10 and Table 5), myricetin (Figure 6) forms six hydrogen bonds with nitrogenous bases of B-DNA (G-12, G-4 and G-2) resulting in more negative Hex binding energy (−6.13 kcal/mol). On the other hand, fisetin (Figure 7) forms only four hydrogen bonds with B-DNA (A-5, G-4 and C-11) as compared to myricetin. This lesser hydrogen bonding resulted in lower negative binding energy (−5.56 kcal/mol). Quercetin (Figure 8) possesses five hydroxyl groups, less than myricetin and greater than fisetin, but it shows the formation of only three hydrogen bonds with nitrogenous bases of B-DNA (G-2, G-4 and G-10). The reason for this may be that a larger portion of the quercetin molecule lies away from the minor groove, as preventing the proximity of the majority of its hydroxyl groups to DNA nitrogenous bases as revealed by docking studies (Figure 8B) hence, relatively less binding energy is obtained. Kaempferol (Figure 9) and galangin (Figure 10) form only two and one hydrogen bond(s) with nitrogenous bases (A-5 and C-9) and (G-10) respectively, resulting in even lower binding energy (KL = −5.24 kcal/mol, GN = −5.01 kcal/mol) as compared to myricetin. Therefore, formation of only one hydrogen bond with nitrogenous bases (A-6 and A-7) of B-DNA, confirms the least stability of galangin with B-DNA amongst all five flavonoids. Again, these results are in conformity with the relative cellular DNA breakage capacity by the five flavonoids used.

**Table 5:** Hydrogen bonds and binding energy data obtained from molecular docking procedure of five docked ligands with B-DNA.

Compounds	Binding Energy (kcal/mol)	Number of Hydrogen Bonds
Myricetin	−6.13	6
Fisetin	−5.56	4
Quercetin	−5.55	3
Kaempferol	−5.24	2
Galangin	−5.01	1

The principle conclusions of the above experiments may be stated as follows: (i) The number and positions of hydroxyl groups in the flavonoid skeleton (Figure 1a) is important in determining the degree of cellular DNA breakage. Solid double headed arrows in Figure 1a also indicate the three most probable group pairs that can form chelates with copper (3–4, 4–5 and 3’–4’) [44]. For stereoelectronic reasons, a **4**–**5** complex cannot be formed from a **3**–**4** complex; (ii) In addition to the above three copper complexing positions, the other hydroxyl groups in the skeleton may also contribute to the DNA breakage capability as these may also form a quinone-like structure once inside the cell. All this will likely contribute to the generation of H_2_O_2_ in the cytoplasm as well as in the nucleus. In this context, it is important not to forget the permeability of the nuclear pore complex to small molecules [45]. It is also to be noted that the degree of fluorescence enhancement (Figure 2) of various flavonoids upon binding to DNA follows the same pattern as the capacity of flavonoids to reduce Cu(II) (Figure 3); (iii) The results also indicate the importance of ortho-dihydroxy groups in the B-ring (such as 3’–4’ or 4’–5’) in myricetin, fisetin and quercetin-mediated effects on cellular DNA degradation as well as in the generation of H_2_O_2_ (Figure 4). This is confirmed by the fact that galangin which does not have these hydroxyls is the least effective in cellular DNA breakage; (iv) Another point of interest is the fact that although quercetin has more hydroxyl groups (5) than kaempferol (4), there does not appear to be much difference in their DNA degrading capacity or Cu(II) reduction. Further, fisetin having only four hydroxyls is more effective than quercetin. A possible reason may be that a larger portion of the quercetin molecule is positioned away from the minor groove (Figure 8B). The relative degree of cellular DNA breakage by five flavonoids is also confirmed by the binding energy and the number of hydrogen bonds that can be formed with DNA as indicated by molecular docking studies (Table 5). Thus, the most important conclusion of our results is that in the A-ring system 3–4 or 4–5 positions and in the B-ring two ortho hydroxyls should be available for complexing of DNA bound copper ions. This unique complexing contributes to generation of reactive oxygen species close to the DNA molecule.

Flavonoids are amphipathic molecules that are capable of partitioning into lipid bilayers. It has been reported that the degree of hydroxylation is an important factor for their cell permeability [46]. Although all classes of plant-derived polyphenols including flavonoids are cytotoxic to cancer cell lines, their *in vivo* effectiveness is limited by their low bioavailability. It has been reported that hydrophobic flavonoids are better absorbed in the intestine and this is supported by the relative ease of permeation of flavonoids through the cell membrane phospholipid bilayer [35]. On the other hand, according to our hypothesis (see Introduction) and as seen in our results, the number of hydroxyl groups in the flavonoid skeleton has a profound effect on flavonoid-induced cytotoxicity. The cytotoxicity of flavonoids is enhanced with the increase in the number of hydroxyl groups, which also leads to an increased hydrophilic nature. Strategies to enhance flavonoid bioavailability as well as their cytotoxicity include incorporation of flavonoid in borneol/methanol eutectic mixtures, microemulsions, polyvinylpyrrilidone dispersion and complexation with cyclodextrin and lecithin [47], in addition to the flavonoid aglycone incorporation into nano crystals [48].

Until now the major emphasis on anticancer properties of flavonoids has been on their chemopreventive effect. Few studies exist on their therapeutic effect in tumor bearing animal models. However, the flavonoid myricetin was shown to be as effective as vincristine as a protective agent in the induction of breast cancer in female Wistar rats [12]. It is worth pointing out that since the intracellular levels of copper are relatively high in cancer cells, amounts of flavonoid(s) required for the anticancer activity in cancer cells would probably be lower, as compared to normal epithelial cells. In support of such an argument, it has indeed been shown that apoptosis could be induced in adenocarcinoma prostate cancer cells (CA-HPV10 cells) by 10 µM concentration of apigenin whereas the normal cells were refractory to even 20 µM concentration of apigenin [49].

## 3. Experimental Section

Myricetin (MN), fisetin (FN), quercetin (QN), kaempferol (KL), galangin (GN), tannic acid, neocuproine, bathocuproinedisulphonate, agarose, Triton X-100, Trypan blue, superoxide dismutase (SOD), RPMI 1640, Histopaque 1077, and phosphate buffered saline (PBS) were purchased from Sigma (St. Louis, MO, USA). All other chemicals were of analytical grade. Fresh solutions of MN, FN, QN, KL and GN were always prepared as 3.0 mM stock in DMSO before use as a 1 mM stock solution. When added to the experimental assays, all the tested flavonoids remained in solution, and the addition of flavonoid stock solutions did not cause any change in the pH of experimental mixtures.

### 3.1. Isolation of Lymphocytes

Heparinized blood samples (2 mL) were collected from a healthy donor through venipuncture and diluted in Ca^2+^/Mg^2+^ free PBS. Histopaque 1077 (Sigma, St. Louis, MO, USA) was used to isolate lymphocytes from blood and, once collected, lymphocytes were suspended in RPMI 1640. All experiments were conducted using blood from the same donor, in order to minimize variation between individual assays.

### 3.2. Viability Assessment of Lymphocytes

The viability of lymphocytes is critical and this was ensured by conducting trypan blue exclusion assay at the beginning and end of each individual assay [50].

### 3.3. Lymphocytes Treatment and Assay for DNA Breakage Using Alkaline Single-Cell Gel Electrophoresis (Comet Assay)

We diluted lymphocytes isolated from a total of 2 mL blood to a count of 2 × 10^5^ cells/2 mL using RPMI 1640. Ten thousand cells were then mixed with 75 µL low melting point agarose (pre-warmed) in PBS. The mixture was promptly applied onto a frosted microscopic slide layered with 75 µL of 1% standard agarose in PBS. Slides were allowed to cool down and gel at 4 °C for 10 min. These lymphocytes on slides were exposed to different flavonoids, as needed for individual assays, and comet assay was performed as described in our earlier studies [5]. In addition to intact lymphocytes, we also permeabilized lymphocytes for individual assays [41]. Such permeabilization was achieved by exposing cells, for 10 min on ice, to the permeabilization solution that consisted of 0.5% Triton X-100 in 0.004 M Tris–HCl, pH = 7.4. Permeabilized cells were treated with test flavonoids, as described for individual assays. For such treatments with individual flavonoids, slides with permeabilized lymphocytes were transferred to a rectangular dish (8 cm × 3 cm × 5 mm) and all the compounds, scavengers, inhibitors *etc.* were added to the dish, as needed. Incubation was done at 37 °C for different time periods: 1 h for whole cells, and 30 min for permeabilized cells. Slides were washed twice in 0.4 M phosphate buffer (pH = 7.5) for 5 min at room temperature before further processing for comet assay.

### 3.4. Fluorescence Studies

Shimadzu spectrofluorometer RF-5310 PC (Kyoto, Japan) was used for all the fluorescence studies. Flavonoids (in 10 mM Tris–HCl, pH = 7.5) were excited at different wavelengths (MN = 380 nm, FN = 365 nm, QN = 370 nm, KL = 365 nm, GN = 365 nm) in the presence of increasing native DNA base pair molar (1:1) and the emission spectra were recorded, as reported in the individual panels.

### 3.5. Stoichiometric Titration of Cu(I) Production

For the measurement of stoichiometry of Cu(I) production, test flavonoids (10 µM) in 10 mM Tris–HCl (pH = 7.5) were mixed with indicated amounts of CuCl_2_ (2.5–70 µM) and a bathocuproine stock solution. Cu(I) forms a complex with bathocuproine that absorbs maximally at 480 nm and, therefore, absorbance at 480nm was recorded after an hour long of incubation in the dark at 37 °C, as described previously [51].

### 3.6. Detection of H_2_O_2_ in the Incubation Medium by FOX Assay

We adapted the FOX assay [42] for the detection and quantification of H_2_O_2_. Our reaction mixture consisted of an incubation medium (RPMI 1640, phosphate buffer 0.4 M, pH = 7.5) and the test flavonoids. This assay involves an oxidation of Fe^2+^ ions to Fe^3+^ ions by H_2_O_2_ with a subsequent binding of generated Fe^3+^ ions to a dye (xylenol orange) which is ferric-sensitive. This binding yields an orange-purple complex, which can be detected at 560 nm. The assay was carried out by incubating the reaction mixtures and the cells for 2 h at 37 °C. At the end of reaction, 200 µL aliquot were analyzed for the detection of H_2_O_2_ [5].

### 3.7. Isothermal Titration Calorimetric Measurements (ITC)

ITC was performed using VP-ITC titration microcalorimeter (MicroCal Inc., Northampton, MA, USA) for the determination of binding energetics of flavonoids to calf thymus DNA (ctDNA) at 25 °C. To avoid the formation of bubbles in the calorimeter cell, flavonoids and ctDNA solutions were degassed prior to assay. Calorimeter and reference cells were loaded with 1.4 mL ctDNA and 1× TE buffer (pH = 8.0), respectively. ctDNA (0.057 mM) was titrated by flavonoids MN, FN, QN, KL, GN (0.4 mM) by up to 28 successive automatic injections of 10 µL each. The individual injection of flavonoid solution was injected into calorimeter cell over 20 s with an interval of 180 s between injections. The reference power set at 16 µcal·s^−1^. Integration of peaks corresponding to each injection as well as the baseline corrections were done using vendor-provided MicroCal Analyzer software (GE healthcare life sciences, Hong Kong, China). Data were fitted and analyzed with one binding site model using Origin 7.0 (Origin Lab Corporation, Northampton MA, USA) to determine equilibrium binding constant (*K*_b_), entropy change and standard enthalpy change of complex formation (∆*H*°).

Other thermodynamic parameters were calculated using the following equations:

∆*G*° = −R*T* ln*K*_b_ (1)

∆*G*° = ∆*H*° − *T*∆*S*° (2)

### 3.8. Molecular Docking Studies

We used HEX 8.0.0 software [52] to study molecular docking. This is an interactive molecular graphic program. Crystal structure of B-DNA dodecamer (CGCGAATTCGCG)_2_ (PDB ID: 1BNA) was downloaded from the protein data bank [53]. The source of flavonoid molfiles was [54]. Avogadro’s 1.01 [55] was used to convert files into PDB format [56] and the Hex 8.0.0 finalized docking using Spherical Polar Fourier Correlations. The parameters that we used for docking were: shape only correlation type, 3D FFT mode, 0.6 grid dimension, 180 receptor range, 180 ligand range, 360 twist range and 40 distance range. PyMol software (DeLano Scientific, San Carlos, CA, USA) was used to visualize docking poses.

### 3.9. Statistics

Statistical analysis was performed as described by Tice [57]. All the values are expressed as ±SEM, and are representative of at least three independent observations. Statistically significant differences were evaluated by using student *t*-test and analysis of variance was performed using ANOVA. *p*-values < 0.05 were considered statistically significant.

## 4. Conclusions

It may therefore be concluded that phytochemicals like flavonoids possess anticancer activity. Myricetin possesses the highest chemopreventive cytotoxic ability among the above reported compounds. The novel information from this work can possibly be utilized for the synthesis and further development of novel molecules with anticancer cytotoxicity and enhanced bioavailability. Therefore, the chemical basis for the differential cytotoxicity of the flavonoids might be attributed to the structural aspects of these compounds.

## References

[1] Rogers A.E., Zeisel S.H., Groopman J. (1993). Diet and carcinogenesis. Carcinogenesis.

[2] Thomasset S.C., Berry D.P., Garcea G., Marczylo T., Steward W.P., Gescher A.J. (2007). Dietary polyphenolic phytochemicals—Promising cancer chemopreventive agents in humans? A review of their clinical properties. Int. J. Cancer.

[3] Ullah M.F., Shamim U., Hanif S., Azmi A.S., Hadi S.M. (2009). Cellular DNA breakage by soy isoflavone genistein and its methylated structural analogue biochanin A. Mol. Nutr. Food Res..

[4] Duo J., Ying G.G., Wang G.W., Zhang L. (2012). Quercetin inhibits human breast cancer cell proliferation and induces apoptosis via bcl-2 and bax regulation. Mol. Med. Rep..

[5] Shamim U., Hanif S., Ullah M.F., Azmi A.S., Bhat S.H., Hadi S.M. (2008). Plant polyphenols mobilize nuclear copper in human peripheral lymphocytes leading to oxidatively generated DNA breakage: Implications for an anticancer mechanism. Free Radic. Res..

[6] Hendrich A.B. (2006). Flavonoid-membrane interactions: Possible consequences for biological effects of some polyphenolic compounds. Acta Pharmacol. Sin..

[7] Cushnie T.P., Lamb A.J. (2011). Recent advances in understanding the antibacterial properties of flavonoids. Int. J. Antimicrob. Agents.

[8] Kumar S., Pandey A.K. (2013). Chemistry and biological activities of flavonoids: An overview. Sci. World J..

[9] Wattel A., Kamel S., Mentaverri R., Lorget F., Prouillet C., Petit J.P., Fardelonne P., Brazier M. (2003). Potent inhibitory effect of naturally occurring flavonoids quercetin and kaempferol on *in vitro* osteoclastic bone resorption. Biochem. Pharmacol..

[10] Wattel A., Kamel S., Prouillet C., Petit J.P., Lorget F., Offord E., Brazier M. (2004). Flavonoid quercetin decreases osteoclastic differentiation induced by RANKL via a mechanism involving NF κB and AP-1. J. Cell. Biochem..

[11] Kuntz S., Wenzel U., Daniel H. (1999). Comparative analysis of the effects of flavonoids on proliferation, cytotoxicity, and apoptosis in human colon cancer cell lines. Eur. J. Nutr..

[12] Jayakumar J.K., Nirmala P., Praveen Kumar B.A., Kumar A.P. (2014). Evaluation of protective effect of myricetin, a bioflavonoid in dimethyl benzanthracene-induced breast cancer in female wistar rats. South. Asian J. Cancer.

[13] Sak K. (2014). Cytotoxicity of dietary flavonoids on different human cancer types. Pharmacogn. Rev..

[14] Nijveldt R.J., van Nood E., van Hoorn D.E., Boelens P.G., van Norren K., van Leeuwen P.A. (2001). Flavonoids: A review of probable mechanisms of action and potential applications. Am. J. Clin. Nutr..

[15] Ahmad M.S., Fazal F., Rahman A., Hadi S.M., Parish J.H. (1992). Activities of flavonoids for the cleavage of DNA in the presence of Cu(II): Correlation with generation of active oxygen species. Carcinogenesis.

[16] Azmi A.S., Bhat S.H., Hanif S., Hadi S.M. (2006). Plant polyphenols mobilize endogenous copper in human peripheral lymphocytes leading to oxidative DNA breakage: A putative mechanism for anticancer properties. FEBS Lett..

[17] Ullah M.F., Ahmad A., Khan H.Y., Zubair H., Sarkar F.H., Hadi S.M. (2013). The prooxidant action of dietary antioxidants leading to cellular DNA breakage and anticancer effects: Implications for chemotherapeutic action against cancer. Cell Biochem. Biophys..

[18] Kagawa T.F., Geierstanger B.H., Wang A.H., Ho P.S. (1991). Covalent modification of guanine bases in double-stranded DNA. The 1.2-a z-DNA structure of d(CGCGCG) in the presence of CuCl_2_. J. Biol. Chem..

[19] Prochazkova D., Bousova I., Wilhelmova N. (2011). Antioxidant and prooxidant properties of flavonoids. Fitoterapia.

[20] Alrawaiq N.S., Abdullah A. (2014). A review of flavonoid quercetin: Metabolism, bioactivity and antioxidant properties. Int. J. Pharm. Tech. Res..

[21] Cao G., Sofic E., Prior R.L. (1997). Antioxidant and prooxidant behavior of flavonoids: Structure-activity relationships. Free Radic. Biol. Med..

[22] Hadi S.M., Asad S.F., Singh S., Ahmad A. (2000). Putative mechanism for anticancer and apoptosis-inducing properties of plant-derived polyphenolic compounds. IUBMB Life.

[23] Bryan S.E. (1979). Metal Ions in Biological Systems.

[24] Burkitt M.J., Milne L., Nicotera P., Orrenius S. (1996). 1,10-Phenanthroline stimulates internucleosomal DNA fragmentation in isolated rat-liver nuclei by promoting the redox activity of endogenous copper ions. Biochem. J..

[25] Ebadi M., Swanson S. (1988). The status of zinc, copper, and metallothionein in cancer patients. Prog. Clin. Biol. Res..

[26] Margalioth E.J., Udassin R., Cohen C., Maor J., Anteby S.O., Schenker J.G. (1987). Serum copper level in gynecologic malignancies. Am. J. Obstet. Gynecol..

[27] Yoshida D., Ikeda Y., Nakazawa S. (1993). Quantitative analysis of copper, zinc and copper/zinc ratio in selected human brain tumors. J. Neuro Oncol..

[28] Ebara M., Fukuda H., Hatano R., Saisho H., Nagato Y., Suzuki K., Nakajima K., Yukawa M., Kondo F., Nakayama A. (2000). Relationship between copper, zinc and metallothionein in hepatocellular carcinoma and its surrounding liver parenchyma. J. Hepatol..

[29] Leist M., Jaattela M. (2001). Four deaths and a funeral: From caspases to alternative mechanisms. Nat. Rev. Mol. Cell Biol..

[30] Ahmad A., Farhan Asad S., Singh S., Hadi S.M. (2000). DNA breakage by resveratrol and Cu(II): Reaction mechanism and bacteriophage inactivation. Cancer Lett..

[31] Khan H.Y., Zubair H., Ullah M.F., Ahmad A., Hadi S.M. (2011). Oral administration of copper to rats leads to increased lymphocyte cellular DNA degradation by dietary polyphenols: Implications for a cancer preventive mechanism. Biometals.

[32] Ullah M.F., Ahmad A., Zubair H., Khan H.Y., Wang Z., Sarkar F.H., Hadi S.M. (2011). Soy isoflavone genistein induces cell death in breast cancer cells through mobilization of endogenous copper ions and generation of reactive oxygen species. Mol. Nutr. Food Res..

[33] Zubair H., Khan H.Y., Sohail A., Azim S., Ullah M.F., Ahmad A., Sarkar F.H., Hadi S.M. (2013). Redox cycling of endogenous copper by thymoquinone leads to ros-mediated DNA breakage and consequent cell death: Putative anticancer mechanism of antioxidants. Cell Death Dis..

[34] Zubair H., Khan H.Y., Ullah M.F., Ahmad A., Wu D., Hadi S.M. (2012). Apogossypolone, derivative of gossypol, mobilizes endogenous copper in human peripheral lymphocytes leading to oxidative DNA breakage. Eur. J. Pharm. Sci..

[35] Gonzales G.B., Smagghe G., Grootaert C., Zotti M., Raes K., Van Camp J. (2015). Flavonoid interactions during digestion, absorption, distribution and metabolism: A sequential structure-activity/property relationship-based approach in the study of bioavailability and bioactivity. Drug Metab. Rev..

[36] Ostling O., Johanson K.J. (1984). Microelectrophoretic study of radiation-induced DNA damages in individual mammalian cells. Biochem. Biophs. Res. Commun..

[37] Singh N.P., McCoy M.T., Tice R.R., Schneider E.L. (1988). A simple technique for quantitation of low levels of DNA damage in individual cells. Exp. Cell Res..

[38] Hanif S., Shamim U., Ullah M.F., Azmi A.S., Bhat S.H., Hadi S.M. (2008). The anthocyanidin delphinidin mobilizes endogenous copper ions from human lymphocytes leading to oxidative degradation of cellular DNA. Toxicology.

[39] Cemeli E., Schmid T.E., Anderson D. (2004). Modulation by flavonoids of DNA damage induced by estrogen-like compounds. Environ. Mol. Mutagen..

[40] Badwey J.A., Karnovsky M.L. (1980). Active oxygen species and the functions of phagocytic leukocytes. Annu. Rev. Biochem..

[41] Czene S., Tiback M., Harms-Ringdahl M. (1997). pH-Dependent DNA cleavage in permeabilized human fibroblasts. Biochem. J..

[42] Long L.H., Clement M.V., Halliwell B. (2000). Artifacts in cell culture: Rapid generation of hydrogen peroxide on addition of (−)-epigallocatechin, (−)-epigallocatechin gallate, (+)-catechin, and quercetin to commonly used cell culture media. Biochem. Biophs. Res. Commun..

[43] Bhat R., Hadi S.M. (1994). DNA breakage by tannic acid and Cu(II): Sequence specificity of the reaction and involvement of active oxygen species. Mutat. Res..

[44] Rahman A., Shahabuddin, Hadi S.M., Parish J.H., Ainley K. (1989). Strand scission in DNA induced by quercetin and Cu(II): Role of Cu(I) and oxygen free radicals. Carcinogenesis.

[45] Mazzanti M. (1998). Ion permeability of the nuclear envelope. Physiological.

[46] Tammela P., Laitinen L., Galkin A., Wennberg T., Heczko R., Vuorela H., Slotte J.P., Vuorela P. (2004). Permeability characteristics and membrane affinity of flavonoids and alkyl gallates in Caco-2 cells and in phospholipid vesicles. Arch. Biochem. Biophys..

[47] Thilakarathna S.H., Rupasinghe H.P. (2013). Flavonoid bioavailability and attempts for bioavailability enhancement. Nutrients.

[48] Li Y., Sun S., Chang Q., Zhang L., Wang G., Chen W., Miao X., Zheng Y. (2013). A strategy for the improvement of the bioavailability and antiosteoporosis activity of bcs iv flavonoid glycosides through the formulation of their lipophilic aglycone into nanocrystals. Mol. Pharm..

[49] Gupta S., Afaq F., Mukhtar H. (2001). Selective growth-inhibitory, cell-cycle deregulatory and apoptotic response of apigenin in normal *versus* human prostate carcinoma cells. Biochem. Biophys. Res. Commun..

[50] Pool-Zobel B.L., Guigas C., Klein R., Neudecker C., Renner H.W., Schmezer P. (1993). Assessment of genotoxic effects by lindane. Food Chem. Toxicol..

[51] Asad S.F., Singh S., Ahmad A., Khan N.U., Hadi S.M. (2001). Prooxidant and antioxidant activities of bilirubin and its metabolic precursor biliverdin: A structure-activity study. Chem. Biol. Interact..

[52] Hex Protein Docking. http://hex.loria.fr/hex.php.

[53] RSCB Protein Data Bank. http://www.rcsb.org./pdb/home/home.do.

[54] The PubChem Project. https://pubchem.ncbi.nlm.nih.gov/.

[55] Avogadro 1.0.1. http://freesoftware.site/download/avogadro-101-10749443.html.

[56] Hanwell M.D., Curtis D.E., Lonie D.C., Vandermeersch T., Zurek E., Hutchison G.R. (2012). Avogadro: An advanced semantic chemical editor, visualization, and analysis platform. J. Cheminform..

[57] Tice R.R., Agurell E., Anderson D., Burlinson B., Hartmann A., Kobayashi H., Miyamae Y., Rojas E., Ryu J.C., Sasaki Y.F. (2000). Single cell gel/comet assay: Guidelines for *in vitro* and *in vivo* genetic toxicology testing. Environ. Mol. Mutagen..

